# Simultaneous silencing of VEGF and KSP by siRNA cocktail inhibits proliferation and induces apoptosis of hepatocellular carcinoma Hep3B cells

**DOI:** 10.1186/0717-6287-47-70

**Published:** 2014-12-15

**Authors:** Chung Chinh Doan, Long Thanh Le, Son Nghia Hoang, Si Minh Do, Dong Van Le

**Affiliations:** Faculty of Biology, University of Science, Vietnam National University, 227 Nguyen Van Cu Street, Ward 4, District 5, Ho Chi Minh City, Vietnam; Department of Animal Biotechnology, Institute of Tropical Biology, Vietnam Academy of Science and Technology, 9/621 Xa lo Ha Noi Street, Linh Trung Ward, Thu Duc District, Ho Chi Minh City, Vietnam; Department of Immunology, Vietnam Military Medical University, 160 Phung Hung Street, Ha Dong District, Ha Noi City, Vietnam

**Keywords:** Vascular endothelial cell growth factor (VEGF), Kinesin spindle protein (KSP), siRNA cocktail, Proliferation, Apoptosis, Hepatocellular carcinoma

## Abstract

**Background:**

Vascular endothelial growth factor (VEGF) is involved in the growth of new blood vessels that feed tumors and kinesin spindle protein (KSP) plays a critical role in mitosis involving in cell proliferation. Simultaneous silencing of VEGF and KSP, an attractive and viable approach in cancer, leads on restricting cancer progression. The purpose of this study is to examine the therapeutic potential of dual gene targeted siRNA cocktail on human hepatocellular carcinoma Hep3B cells.

**Results:**

The predesigned siRNAs could inhibit VEGF and KSP at mRNA level. siRNA cocktail showed a further downregulation on KSP mRNA and protein levels compared to KSP-siRNA or VEGF-siRNA, but not on VEGF expression. It also exhibited greater suppression on cell proliferation as well as cell migration or invasion capabilities and induction of apoptosis in Hep3B cells than single siRNA simultaneously. This could be explained by the significant downregulation of Cyclin D1, Bcl-2 and Survivin. However, no sigificant difference in the mRNA and protein levels of ANG2, involving inhibition of angiogenesis was found in HUVECs cultured with supernatant of Hep3B cells treated with siRNA cocktail, compared to that of VEGF-siRNA.

**Conclusion:**

Silencing of VEGF and KSP plays a key role in inhibiting cell proliferation, migration, invasion and inducing apoptosis of Hep3B cells. Simultaneous silencing of VEGF and KSP using siRNA cocktail yields promising results for eradicating hepatocellular carcinoma cells, a new direction for liver cancer treatment.

## Background

Primary liver cancer, hepatoblastoma (HB) and hepatocellular carcinoma (HCC), is one of the most common solid tumors, ranking the fifth in most common malignancy worldwide and the second cause of cancer-related deaths. The major therapeutic strategies in solid tumors as well as HCC are excision of the primary tumor, followed by radiotherapy and chemotherapy. However, in some cases, this treatment still leaves some problems such as metastatic reactivation and subsequent tumor recurrence [1]. Recently, following the rapid advances in molecular biology, many new therapeutic strategies, including RNA interference (RNAi) technology for treating liver cancer at genetic level have been developed [2]. RNAi is a specific gene regulatory mechanism in which activation of an intracellular pathway triggered by small-interfering RNA (siRNA) of 21–23 nucleotides (nt), leading to gene silencing through degradation of a homologous target mRNA [3]. The selective and robust effect of RNAi on gene expression makes it become a valuable tool for basic research in biology, and thereby continue to have a major impact on medical science [4]. Another unique advantage of RNAi is that non-druggable protein targets can also be efficiently knocked-down and possibly achieve therapeutic effects [5]. Therefore, RNAi-based therapeutic strategy presents an effective and simple approach in new area of clinical therapy for HCC.

It has been known that human cancer is a gene-related disease involving abnormal cell growth. As a new member of the kinesin superfamily of microtubule-based motors, kinesin Eg5, also called kinesin spindle protein (KSP) or KIF11 participates in mitosis, by separating the microtubules that are attached to the two centrosomes, and contributing to the bipolar arrangement of the spindles [6]. Thus, inhibition of KSP may block the formation of bipolar mitotic spindles of mitotic cells, causing cell-cycle arrest, activation of the mitotic checkpoint, induction of apoptosis and eventually, to cell death [5, 7]. KSP gene was found to be lowly expressed in normal primary cells, but higher in transformed cells . Its expression was also higher in breast, colon, lung, ovary, and uterine carcinomas than in their adjacent tissues [8]. The overexpression of KSP as a transgene may cause genomic instability and tumor formation in mice [9]. In addition, KSP gene was also frequently expressed in HCC tissues and there was also a strong correlation between the level of KSP expression and HCC development [10]. These findings have indicated that the important role of KSP in mitotic progression makes it an significant candidate of anticancer therapy. Several KSP inhibitors have been studied in clinical trials and showed efficacy in preclinical models of human tumors [10, 11]. However, more trials must be studied to test their efficacy in clinic due to the toxicological side effects of KSP inhibitors, such as the observed neutropenia and leukopenia [12].

Additionally, the ability of the highly vascularized tumors, including HCC to attract blood vessels (tumor angiogenesis) is one of the rate-limiting steps for tumor progression [13]. Angiogenesis is governed differently by multiple factors, including growth factors, cytokines, chemokines, enzymes, and adhesion molecules, but the most important one is vascular endothelial growth factor (VEGF) [14]. Among all family members of VEGF, VEGF-A is the most potent and specific angiogenic factor. Many studies have shown that VEGF, mainly VEGF-A, is frequently expressed in HCC and increased VEGF levels correspond to increased tumor sizes [14, 15]. Another study reported that there was also a strong correlation between the level of VEGF expression and HCC pathological grading and clinical stages [16]. In addition, VEGF was identified as a key hypoxia-induced angiogenic stimulator in liver cancer [14]. It was suggested that the gene plays a critical role in the HCC progression of tumor growth. Therefore, VEGF is a logical target for HCC therapy. For the last decade, there have been several options of inhibiting VEGF binding to its receptors which have been developed as anticancer agents, such as soluble VEGF receptors, humanized anti VEGF monoclonal antibody (Bevacizumab; Avastin), various small molecules inhibiting VEGFR2 signal transduction [17, 18]. However, the use of anti VEGF antibodies or other inhibitors is responsible for unexpected toxic side effects, especially in terms of thromboembolic events and bleeding that require further investigation [18]. It is therefore a challenge to explore a new approach to inhibit VEGF expression in identification of novel druggable targets.

In this study, we aimed to use siRNA cocktail which targets VEGF-A (referred here as VEGF) and KSP gene as a therapy for HCC treatment. Pre-designed VEGF and KSP siRNAs were screened in Hep3B cell line, isolated from liver biopsy specimens with primary HCC and widely used as an experimental model. The best siRNA targets were used as cocktail to inhibit the growth, migration, invasion and induce apoptosis of Hep3B cells. The effect of siRNA cocktail on inhibiting *in vitro* angiogenesis ability of HUVECs induced by Hep3B cells was also evaluated.

## Results

### Effects of pre-designed siRNAs on KSP and VEGF mRNA expression in Hep3B cells

To address the functions of VEGF and KSP, Hep3B cells were transfected with VEGF-siRNAs and KSP-siRNAs. Subsequently, the relative mRNA levels were determined by Real-time qRT-PCR after treatments for 72 hours. For validation purposes, three different siRNAs targeting different regions of human VEGF or KSP were employed (Table 1). Then, one with best repressive effect was used in following experiments.

**Table 1:** **Sequences of siRNAs targeting VEGF and KSP**

siRNA	Sequences (5’–3’)
VEGF-siRNA#1	Sense: GCACAUAGGAGAGAUGAGCUUdTdT
	Antisense: AAGCUCAUCUCUCCUAUGUGCUGdTdT
VEGF-siRNA#2	Sense: UGAAGUUCAUGGAUGUCUAdTdT
	Antisense: UAGACAUCCAUGAACUUCAdTdT
VEGF-siRNA#3	Sense: GCCUUGCCUUGCUGCUCUAdTdT
	Antisense: UAGAGCAGCAAGGCAAGGCdTdT
KSP-siRNA #1	Sense: CUGAAGACCUGAAGACAAUdTdT
	Antisense: AUUGUCUUCAGGUCUUCAGdTdT
KSP-siRNA #2	Sense: UCGAGAAUCUAAACUAACUdTdT
	Antisense: AGUUAGUUUAGAUUCUCGAdTdT
KSP-siRNA #3	Sense: CUGGAUCGUAAGAAGGCAGdTdT
	Antisense: CUGCCUUCUUACGAUCCAGdTdT
CONT-siRNA	Sense: GCGGAGAGGCUUAGGUGUAdTdT
	Antisense: UACACCUAAGCCUCUCCGCdTdT

**Figure 1:**
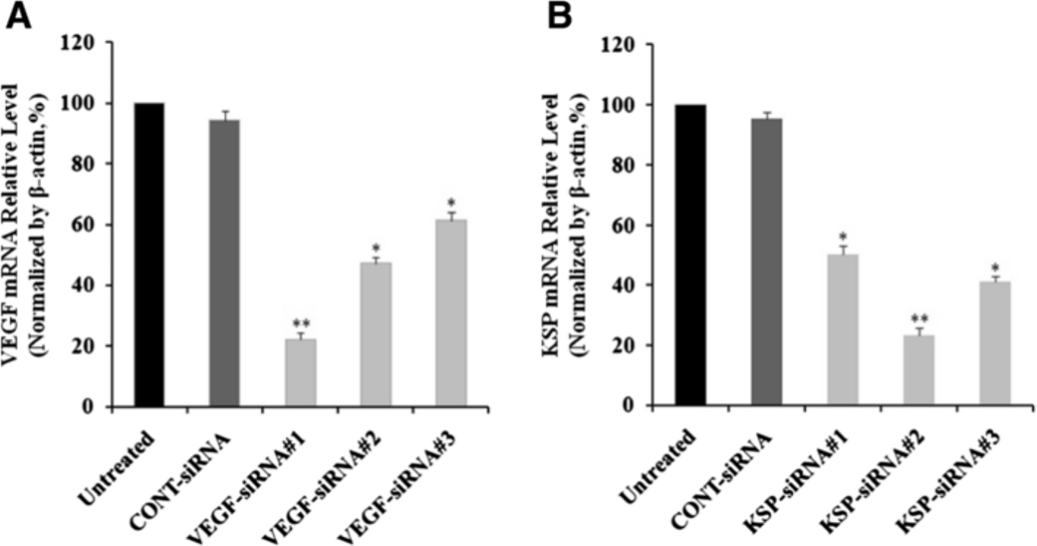
**Effects of pre-designed siRNAs treatments on VEGF and KSP mRNA expression in Hep3B cells.** Cells were transfected with siRNAs. Total RNA was extracted from cells at 72 hours after siRNA transfection. The mRNA relative level of VEGF **(A)** and KSP **(B)** with siRNAs treatments in Hep3B cells by Real-time qRT-PCR. The mRNA expressions of VEGF and KSP were normalized with β-actin. Values were given as mean value ± standard deviation (SD) of triplicate. **p < 0.01 and *p < 0.05 compared to untreated cell group.

As shown in Figure 1A, Real-time qRT-PCR revealed that the inhibition of VEGF expression in the VEGF-siRNA#1, VEGF-siRNA#2, and VEGF-siRNA#3 groups were 77.88 ± 2.02%, 52.68 ± 1.86% and 38.52 ± 2.56% respectively, compared to the untreated group (*p* < 0.05 and *p* < 0.01, Figure 1A). In the same manner, the silencing effects of KSP-siRNAs also observed in the KSP-siRNA#1, KSP-siRNA#2 and KSP-siRNA#3 groups were 49.58 ± 2.64%, 76.72 ± 2.27% and 58.86 ± 1.52%, respectively, compared to the untreated group (*p* < 0.05 and *p* < 0.01, Figure 1B). No significant difference was identified between CONT-siRNA treated cells and control untreated ones. VEGF-siRNA#1 and KSP-siRNA#2, directed at VEGF and KSP, respectively, were selected as the most effective inhibitors for investigation in further experiments.

### Effects of VEGF-siRNA#1, KSP-siRNA#2 and siRNA cocktail on KSP and VEGF expression in Hep3B cells

**Figure 2:**
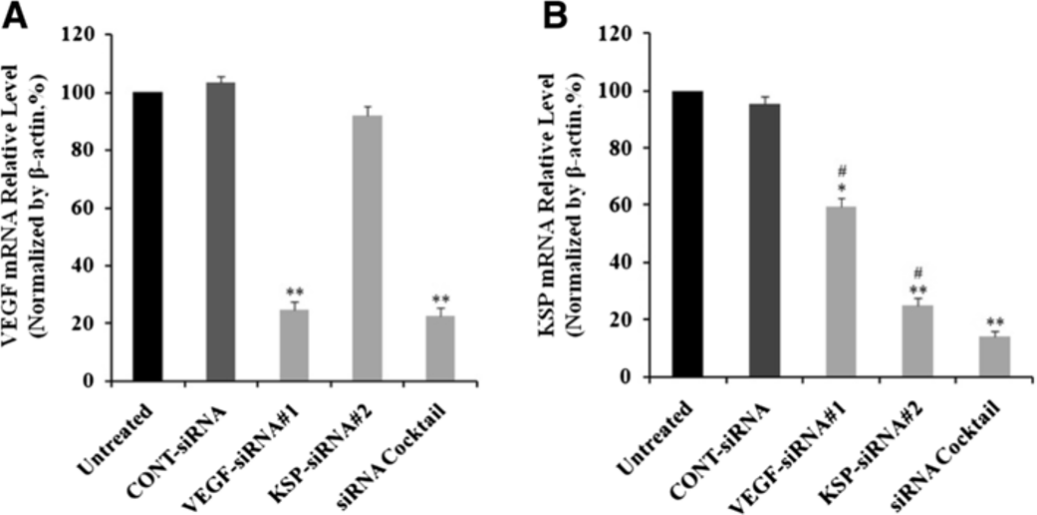
**Effects of different treatments on VEGF and KSP mRNA expression in Hep3B cells.** The mRNA relative level of VEGF **(A)** and KSP **(B)** with different treatments in Hep3B cells by Real-time qRT-PCR. The mRNA expressions of VEGF and KSP were normalized with β-actin. Values were given as mean value ± standard deviation (SD) of triplicate. **p < 0.01, *p < 0.05 compared to untreated cell group and ^#^ *p* < 0.05 compared to siRNA cocktail treated cell group.

**Figure 3:**
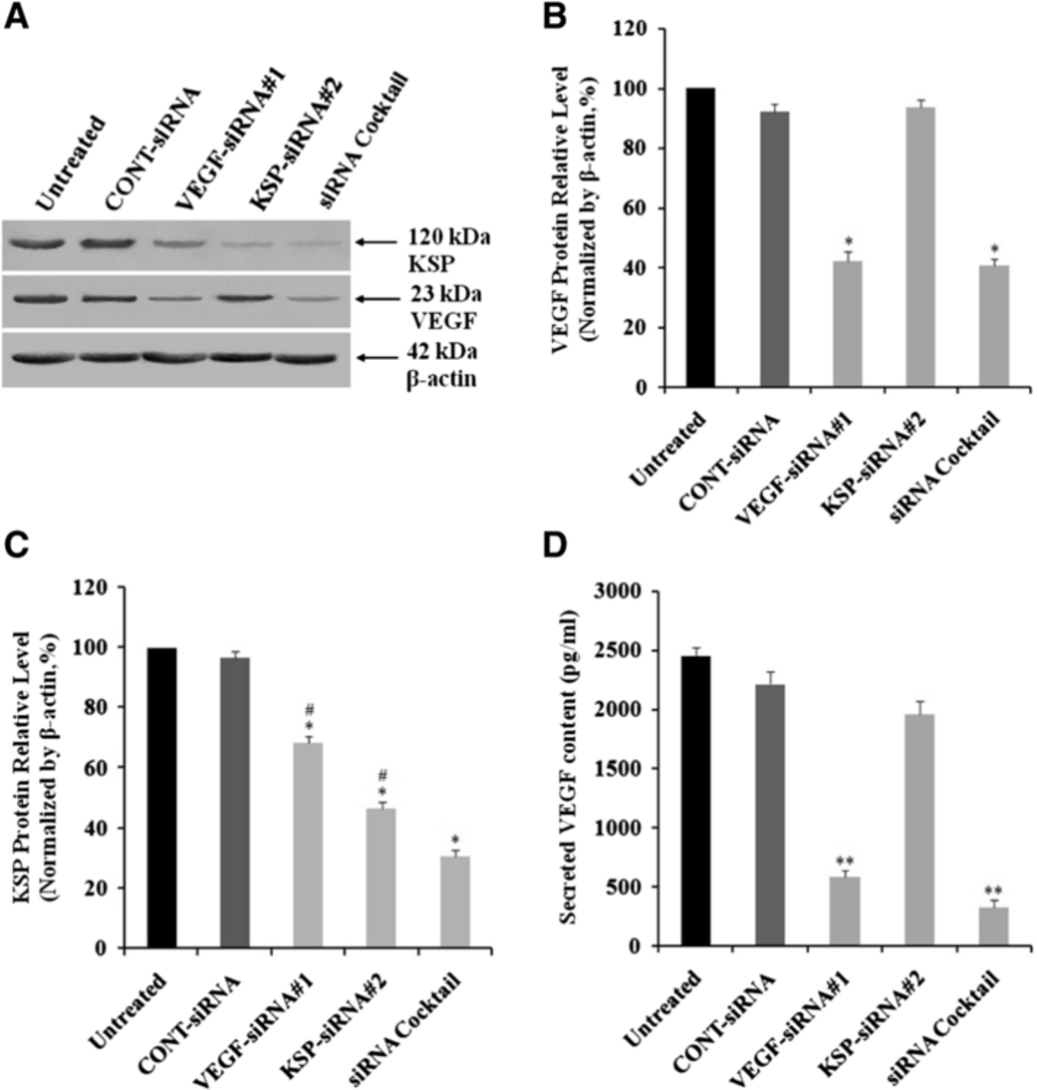
**Effects of different treatments on VEGF and KSP protein expression in Hep3B cells. (A)** The protein expressions of VEGF and KSP were examined by Western blot analyses. β-actin was used as a housekeeping gene control. The size of each protein was indicated. **(B, C)** The siRNAs transfected cells exhibited a decreased expression of VEGF protein **(B)** and KSP protein **(C)** as confirmed by densitometric analysis. **(D)** The cell culture supernatants were collected at 72 hours after transfection and the secreted VEGF concentrations were measured by the quantitative VEGF ELISA kit. Values were given as mean value ± standard deviation (SD) of triplicate. **p < 0.01, *p < 0.05 compared to untreated cell group and ^#^ *p* < 0.05 compared to siRNA cocktail treated cell group.

VEGF-siRNA#1, KSP-siRNA#2, siRNA cocktail (mixed by VEGF-siRNA#1 and KSP-siRNA#2 equally) and CONT-siRNA were transfected into Hep3B cells. The levels of mRNA of VEGF and KSP were determined using Real-time qRT-PCR techniques and protein expression was detected by Western blot and ELISA after treatment with siRNAs for 72 hours. As demonstrated in Figure 2A, VEGF-siRNA#1 inhibited VEGF expression at the mRNA level up to 75.32 ± 3.03%, after 72 hours while it was not much altered in CONT-siRNA transfected cells compared to that of the untreated ones (*p* < 0.01, Figure 2A). A silencing effect of VEGF-siRNA#1 was observed at the protein level up to 57.86 ± 3.35% by Western blot analysis and densitometric analysis (*p* < 0.05, Figure 3A and B). Downregulation of VEGF protein was also confirmed by ELISA analysis (Figure 3D). Interestingly, we found that VEGF was silenced by VEGF-siRNA, but KSP was also inhibited by it at mRNA level up to 40,67 ± 2.96% (*p* < 0.05, Figure 2B), and the detection of protein expression was confirmed by downregulation, protein level up to 31.74 ± 2.38% (*p* < 0.05, Figure 3C) compared to untreated cells.

Similarly, KSP expression was effectively inhibited by KSP-siRNA#2 at both mRNA and protein levels by 75.07 ± 3.56% (*p* < 0.01, Figure 2B) and 53.48 ± 2.19% (*p* < 0.05, Figure 3C) by Real-time qRT-PCR analysis and Western blot analysis, respectively. These values indicated that the effective silencing of KSP-siRNA#2 on both mRNA and protein levels of KSP. As shown in Figures 2 and 3, KSP-siRNA#2 did not produce any effect on the VEGF expression at the mRNA and protein levels.

Eventually, we examined siRNA cocktail on VEGF and KSP expressions respectively. As shown in Figures 2 and 3, siRNA cocktail inhibited the VEGF and KSP expression at the mRNA and protein levels, obviously in comparison to the untreated ones. The results showed that VEGF mRNA was downregulated by 77.54 ± 3.22% (*p* < 0.01, Figure 2A) and VEGF protein level was downregulated by 59.42 ± 2.14% (*p* < 0.05, Figure 3B), which was also confirmed by ELISA analysis compared to untreated cells (Figure 3D). Downregulation of VEGF by siRNA cocktail was similar with that of VEGF-siRNA#1. When compared to VEGF-siRNA#1 or KSP-siRNA#2 alone, the siRNA cocktail showed higher inhibition on KSP mRNA expression up to 85.77 ± 1.78% (*p* < 0.01, Figure 2B) and protein level up to 69.42 ± 2.11% (*p* < 0.05, Figure 3C), indicating a significant effect of siRNA cocktail on KSP expression.

### Effects of VEGF-siRNA#1, KSP-siRNA#2 and siRNA cocktail on cell proliferation in Hep3B cells

**Figure 4:**
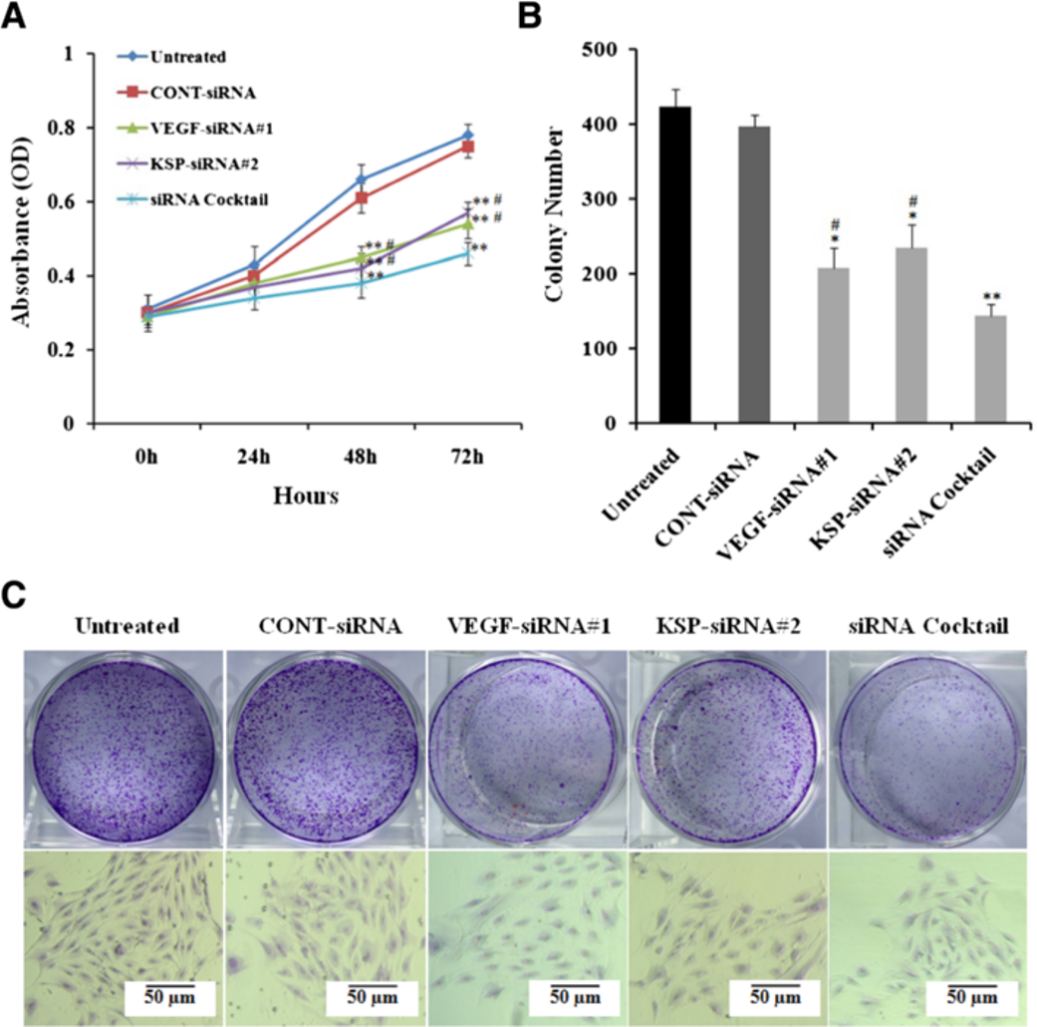
**Effects of different treatments on the growth and the colony formation in Hep3B cells. (A)** The proliferation of Hep3B cells was measured using WST-1 kit. The growth curve of Hep3B cells was shown for each group. The proliferation was assayed in triplicates at 0, 24, 48 and 72 hour post-transfection of siRNAs. **(B)** Effects of different treatments on the inhibition of cell proliferation were confirmed by the total numbers of colony. **(C)** Representative images of the colony formation assay were shown. Values were given as mean value ± standard deviation (SD) of triplicate. **p < 0.01, *p < 0.05 compared to untreated cell group; ^#^ *p* < 0.05 compared to siRNA cocktail treated cell group.

The silencing effects of VEGF and KSP on cell proliferation of Hep3B cells were detected by WST-1 assay and clonogenic survival assay. The absorbance values of the Hep3B cells at 48 and 72 hour post-transfection with siRNA cocktail and either VEGF-siRNA#1 or KSP-siRNA#2 were significantly lower than those of the untreated cells (both *p* < 0.01, Figure 4A). There was no significant difference between the growth of cells treated with VEGF-siRNA#1 and that of KSP-siRNA#2. The absorbance value of Hep3B cells treated with siRNA cocktail showed a significant decrease in cell proliferation compared to the cells treated with either VEGF-siRNA#1 or KSP-siRNA#2 at 48 or 72 hours, respectively (both *p* < 0.05, Figure 4A). These results were also further supported by clonogenic survival assay (Figure 4B). A highly-significant decline of the cloning efficiency was observed in VEGF-siRNA#1 treated group (*p* < 0.05) and KSP-siRNA#2 treated group (*p* < 0.05) as well as siRNA cocktail treated group (*p* < 0.01) in comparison to untreated cells. The inhibition rate treated with siRNA cocktail showed a significant decrease in colony formation compared to the cells treated with either VEGF-siRNA#1 or KSP-siRNA#2 (both *p* < 0.05, Figure 4B and C).

### Effects of VEGF-siRNA#1, KSP-siRNA#2 and siRNA cocktail on cell migration ability in Hep3B cells

**Figure 5:**
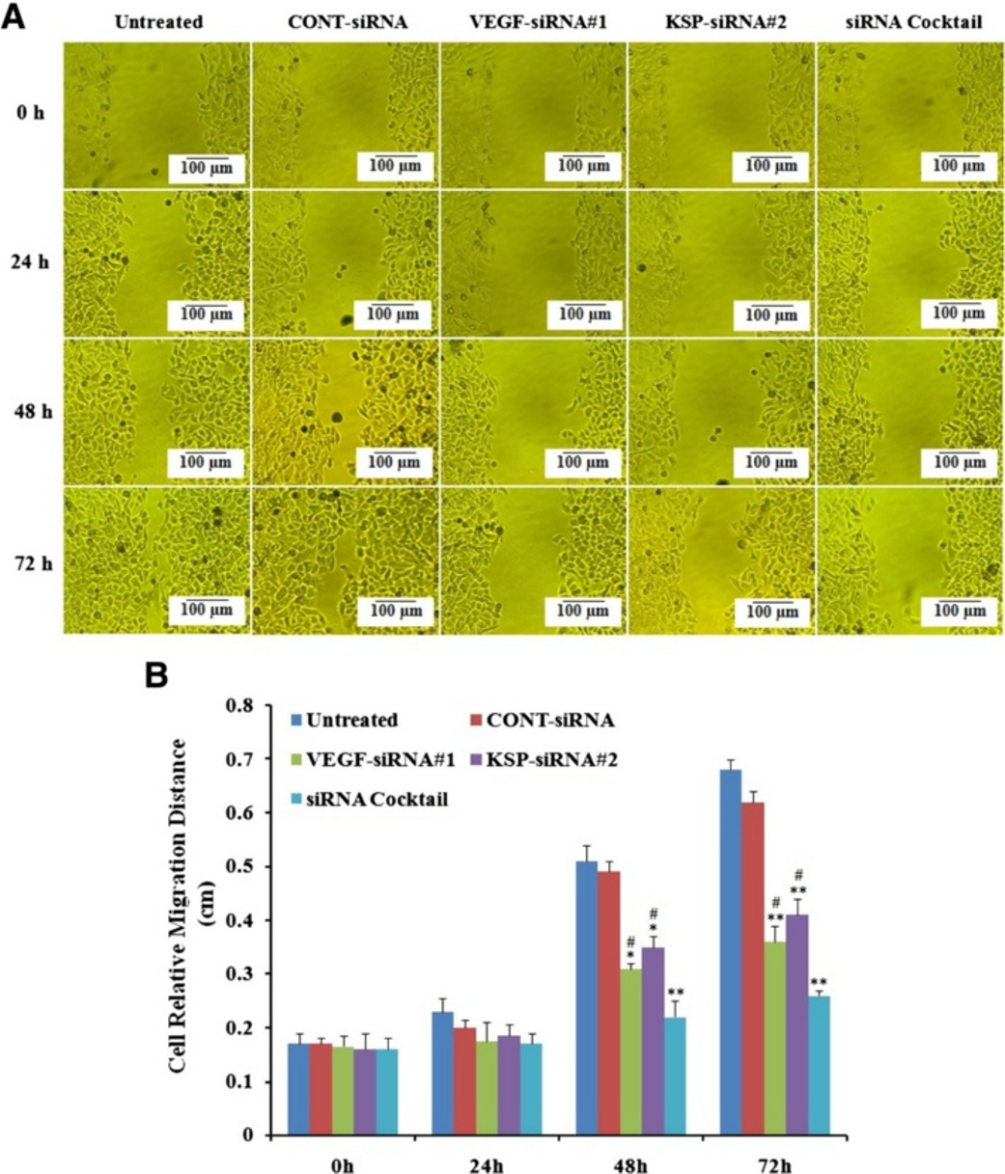
**Effects of different treatments on cell migration in Hep3B cells.** The cells with different treatments at 0, 24, 48, and 72 hours. **(A)** Representative images of the cell migration ability assay were shown. **(B)** Effects of different treatments on migration ability of Hep3B cells were determined by the cell relative migration distances in different time points. Value were presented as mean value ± standard deviation (SD) of triplicate. **p < 0.01, *p < 0.05 compared to untreated cell group and ^#^ *p* < 0.05 compared to siRNA cocktail treated cell group.

Wound-healing assay was used to evaluate the migration ability of Hep3B cells after different treatments. As illustrated in Figure 5A, the scratch caused in groups of untreated and CONT-siRNA nearly closed completely after 72 hours, but the cells in treatment with siRNA cocktail and VEGF-siRNA#1 or KSP-siRNA#2 were not able to move toward the center of the wound. Moreover, siRNA cocktail exhibited a decrease in wound healing ability compared to VEGF-siRNA#1 or KSP-siRNA#1 alone (both *p* < 0.05, Figure 5B).

### Effects of VEGF-siRNA#1, KSP-siRNA#2 and siRNA cocktail on cell invasion ability in Hep3B cells

**Figure 6:**
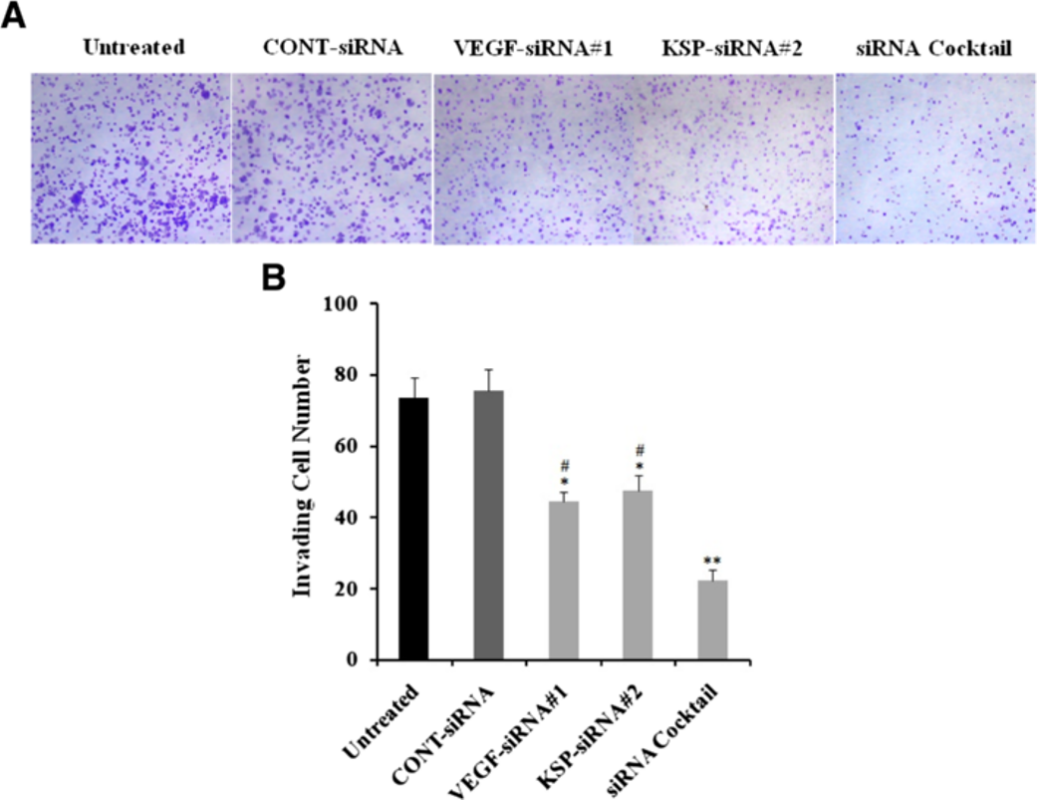
**Effects of different treatments on cell invasion in Hep3B cells.** The cells were treated with different treatments. After 48 hours, cells migrated to the underside of the transwell filters were stained with Crystal Violet solution and imaged. **(A)** Representative images of the cell invasion ability assay were shown. **(B)** Effects of different treatments on invasion ability of Hep3B cells were determined by the total numbers of invading cell. Value were presented as mean value ± standard deviation (SD) of triplicate. **p < 0.01, *p < 0.05 compared to untreated cell group and ^#^ *p* < 0.05 compared to siRNA cocktail treated cell group.

We also performed transwell assay to evaluate the effects of VEGF-siRNA#1, KSP-siRNA#2 and siRNA cocktail on Hep3B cell invasion. Hep3B cells were treated with siRNAs and loaded to the transwell chambers (the upper surface of the transwell filters was coated with matrigel). After 48 hours, cells migrated to the underside of the transwell filters were stained with crystal violet solution and imaged (Figure 6A). As shown in Figure 6, VEGF-siRNA#1, KSP-siRNA#2 and siRNA cocktail significantly suppressed the ability of Hep3B cell to invade to the underside of the transwell filters. And obviously, treatment with siRNA cocktail resulted in a significant decrease of invasion ability compared to that of VEGF-siRNA#1or KSP-siRNA#2 alone treated cells (both *p* < 0.05, Figure 6B).

### Effects of VEGF-siRNA#1, KSP-siRNA#2 and siRNA cocktail on apoptosis in Hep3B cells

**Figure 7:**
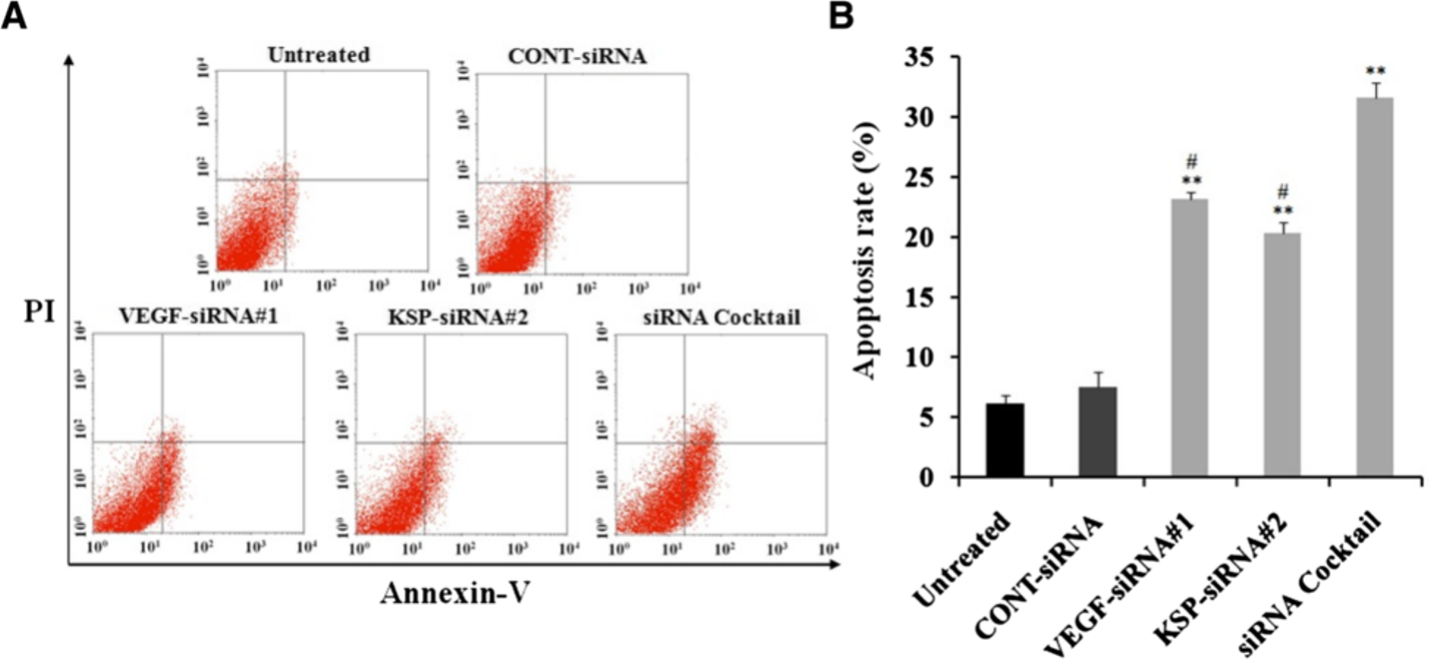
**Effects of different treatments on the induction of apoptosis in Hep3B cells. (A)** Cell apoptosis was detected by Annexin V-FITC/PI double staining and FCM analysis. Cells in the lower left (LL) quadrant represented survivals; lower right (LR) quadrant represented early apoptosis; the upper right (UR) quadrant represented necrosis or post-apoptotic and the upper left (UL) quadrant represented detection of error allowed. **(B)** Values (intensity of fluorescent positive cells during early apoptotic events) were given as mean value ± standard deviation (SD) of triplicate. **p < 0.01 compared to untreated cell group, ^#^ *p* < 0.05 compared to siRNA cocktail treated cell group.

Annexin V-FITC/PI double staining and flow cytometry analysis were performed to evaluate the ability of siRNA cocktail, VEGF-siRNA#1, or KSP-siRNA#2 on inducing Hep3B cell apoptosis. As Figure 7 illustrated, the apoptosis rate of Hep3B cells was significantly increased by VEGF-siRNA#1 treatment (23.25 ± 0.56%) compared to the untreated cells (*p* < 0.01). Similarly, an increase was also identified by KSP-siRNA#2 transfection (20.38 ± 0.89%, *p* < 0.01). In addition, the rate of apoptotic cells were greatly increased by siRNA cocktail treatment (33.62 ± 1.25%, *p* < 0.01). There was no significant difference between the apoptosis rate of the CONT-siRNA treated cells and that of untreated ones. And obviously, treatment with siRNA cocktail resulted in a significant increase of apoptosis compared to that of VEGF-siRNA or KSP-siRNA treated cells (both *p* < 0.05, Figure 7B).

### Inhibition of Cyclin D1, Bcl-2 and Survivin expression in Hep3B cells by VEGF-siRNA#1, KSP-siRNA#2 and siRNA cocktail

**Figure 8:**
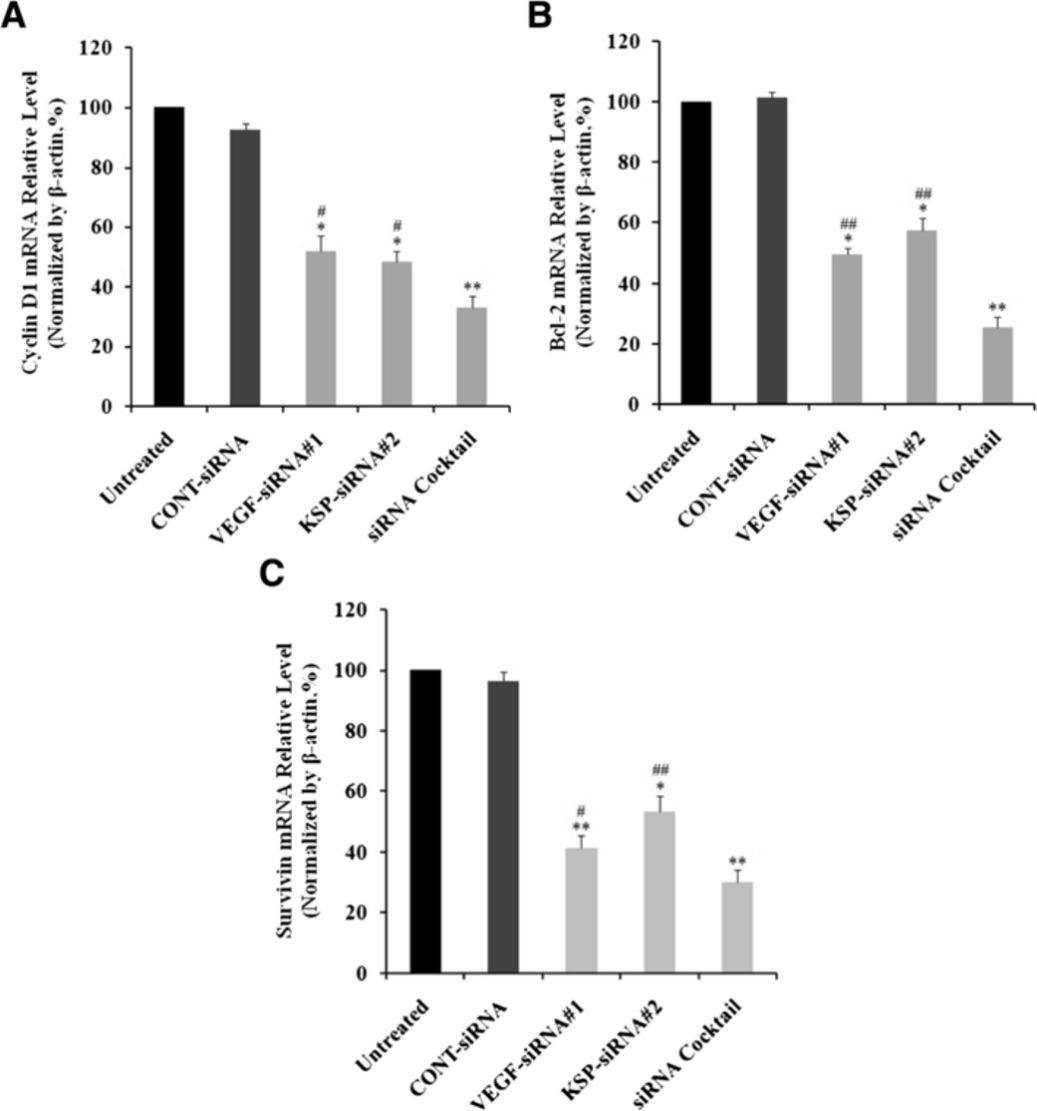
**Effects of different treatments on Cyclin D1, Bcl-2 and Survivin mRNA expression in Hep3B cells.** The mRNA levels of Cyclin D1 **(A)**, Bcl-2 **(B)** and Survivin **(C)** in Hep3B cells were determined by Real-time qRT-PCR after 72 hours of siRNA transfection. The mRNA expression of these genes was normalized with β-actin. Values were given as mean value ± standard deviation (SD) of triplicate. **p < 0.01, *p < 0.05 compared to untreated cell group and ^##^ *p* < 0.01, ^#^ *p* < 0.05 compared to siRNA cocktail treated cell group.

**Figure 9:**
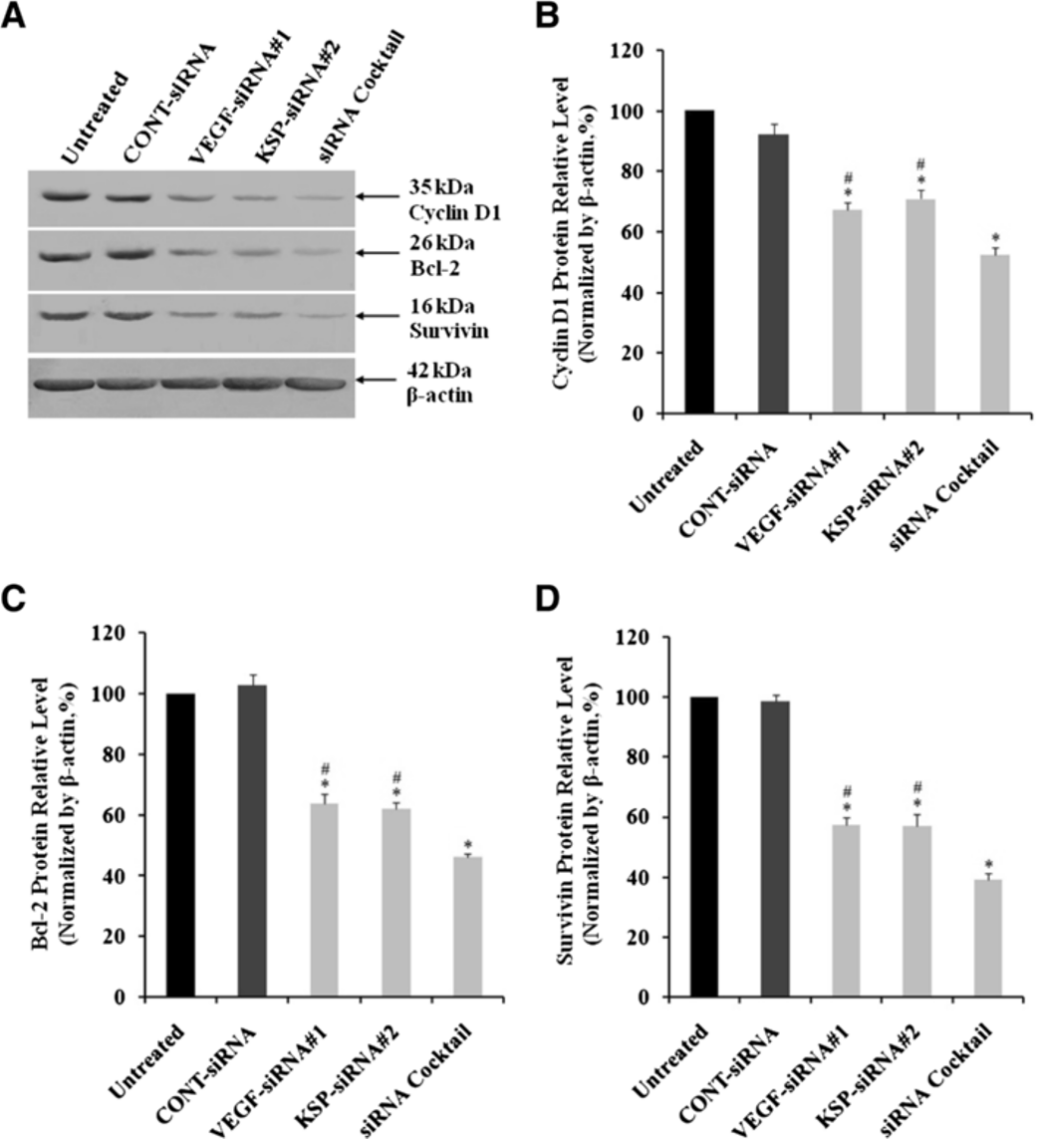
**Effects of different treatments on Cyclin D1, Bcl-2 and Survivin protein expression in Hep3B cells. (A)** The protein expressions of Cyclin D1, Bcl-2 and Survivin in Hep3B cells were measured by Western blot analyses after 72 hours of siRNA transfection. β-actin was used as a housekeeping gene control. The size of each protein was indicated. **(B, C, D)** Densitometric analyses of these three proteins Cyclin D1 **(B)**, Bcl-2 **(C)** and Survivin **(D)** were made relative to β-actin. Values were given as mean value ± standard deviation (SD) of triplicate. *p < 0.05 compared to untreated cell group and ^#^ *p* < 0.05 compared to siRNA cocktail treated cell group.

Downstream targets of Cyclin D1, Bcl-2, and Survivin were also downregulated at both protein and mRNA levels. The relative levels of mRNA of Cyclin D1, Bcl-2 and Survivin were also determined using Real-time RT-qPCR. The mRNA levels of Cyclin D1 and Bcl-2 were downregulated by 48.21 ± 5.02%, 51.77 ± 3.52% and 64.23 ± 4.02% (Figure 8A); 47.57 ± 2.04%, 43.72 ± 4.23% and 60.74 ± 5.02% (Figure 8B), whereas the mRNA levels of Survivin were downregulated by 57.64 ± 4.05%, 55.75 ± 5.03% and 70.12 ± 4.26% (Figure 8C) in VEGF-siRNA#1, KSP-siRNA#2 and siRNA cocktail transfected Hep3B cells in comparison to the untreated cells, respectively (*p* < 0.05 and *p* < 0.01, Figure 8). Similarly, both Cyclin D1, Bcl-2 and Survivin protein expressions were measured by using Western blot analyses (Figure 9A). The protein levels of Cyclin D1 and Bcl-2 were downregulated by 32.62 ± 2.38%, 29.12 ± 3.05% and 45.78 ± 2.54% (Figure 9B); 36.34 ± 3.05%, 38.13 ± 2.19% and 47.92 ± 1.15% (Figure 9C), and Survivin protein expressions were decreased by 42.70 ± 2.56%, 43.05 ± 3.84% and 56.92 ± 2.05% (Figure 9D) in VEGF-siRNA#1, KSP-siRNA#2 and siRNA cocktail transfected Hep3B cells compared to the untreated cells, respectively (*p* < 0.05 and *p* < 0.01, Figure 9). siRNA cocktail showed greater decrease of Cyclin D1, Bcl-2, Survivin expression at both mRNA and protein levels in comparison to VEGF-siRNA#1 or KSP-siRNA#2 alone (*p* < 0.05 and *p* < 0.01, Figures 8 and 9). There was no significant difference in mRNA and protein levels of Cyclin D1, Bcl-2 and Survivin between CONT-siRNA treated cells and untreated ones.

### Effects of VEGF-siRNA#1, KSP-siRNA#2 and siRNA cocktail on tube formation in HUVECs

**Figure 10:**
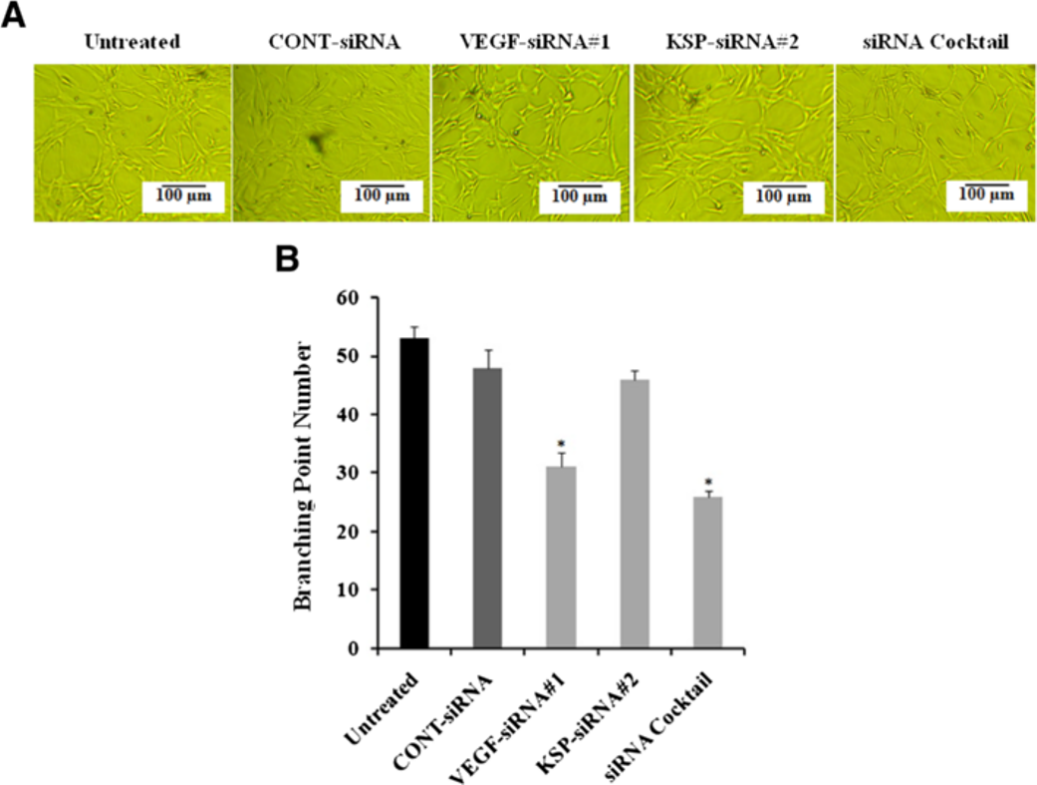
**The tube formation inhibition of different treatments was detected in HUVECs angiogenesis model. (A)** Representative photographs of each treatments were shown. **(B)** The total numbers of branching points were decreased by treatment of siRNA cocktail, VEGF-siRNA#1 and KSP-siRNA#2 compared to the untreated. Values were given as mean value ± standard deviation (SD) of triplicate. *p < 0.05 compared to untreated cell group.

A HUVECs angiogenesis model was employed to evaluate the tube formation of HUVECs stimulated by the conditioned medium derived from Hep3B cells transfected with siRNA cocktail, VEGF-siRNA#1, KSP-siRNA#2 and CONT-siRNA. As illustrated in Figure 10, siRNA cocktail or VEGF-siRNA#1 transfected Hep3B cells inhibited HUVECs to form extensive and enclosed tube networks on Matrigel as compared to the CONT-siRNA treated cells and untreated ones (*p* < 0.05, Figure 10B). However, KSP-siRNA#2 treated cells did not affect on tube formation in HUVECs.

**Figure 11:**
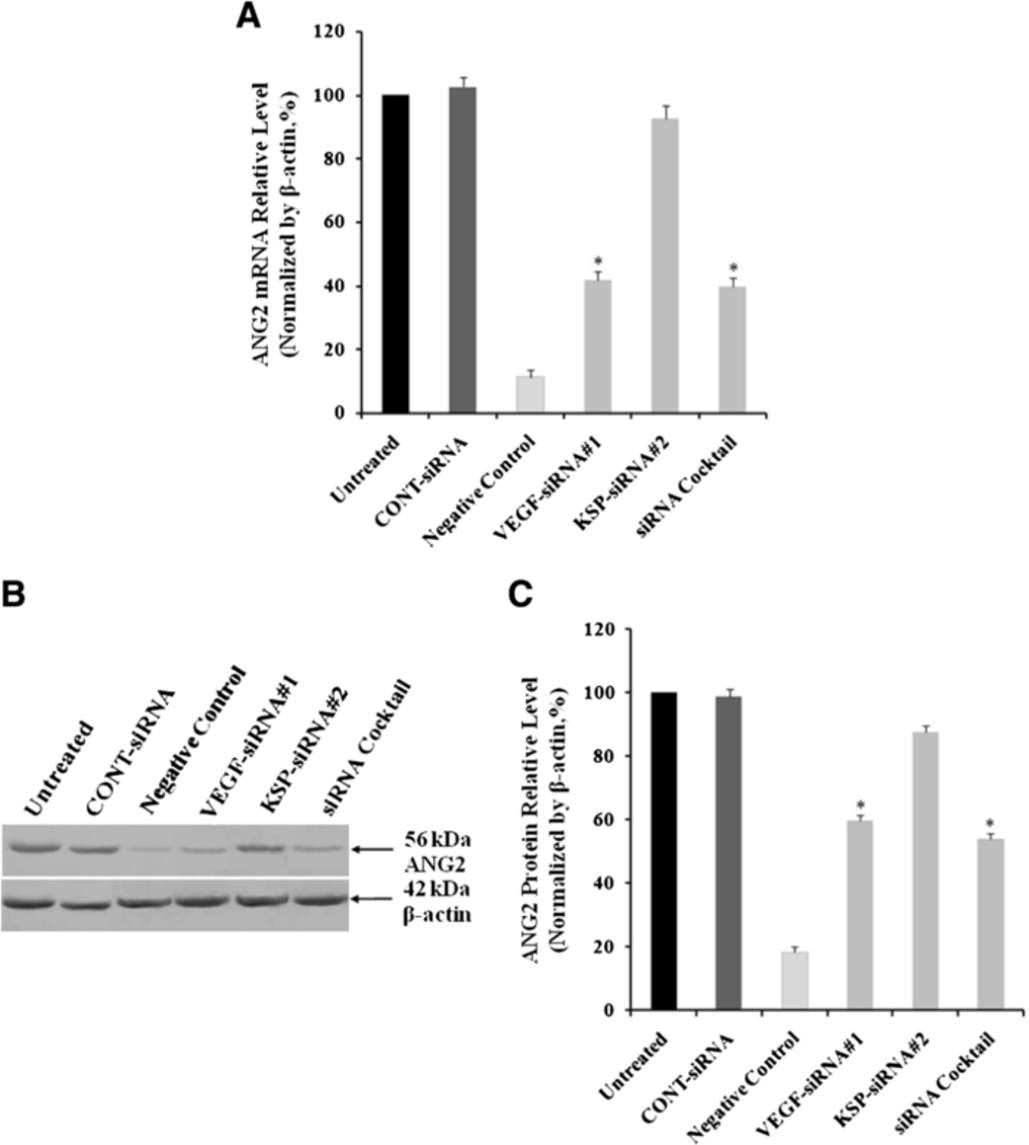
**Effects of different treatments on ANG2 expression in HUVECs cultured with Hep3B supernatant.** HUVEC complete medium (without VEGF) was used as a negative control. **(A)** ANG2 mRNA expression levels were showed by Real-time qRT-PCR analysis. **(B)** ANG2 protein in HUVECs was determined by Western Blot analysis. **(C)** Densitometric analysis of ANG2 protein was made relative to β-actin. Values were given as mean value ± standard deviation (SD) of triplicate. *p < 0.05 compared to untreated cell group.

We also determined the mRNA and protein levels of ANG2 in HUVECs. In normally cultured negative control cells, the expression of ANG2 mRNA (11.24 ± 2.15%) and protein (18.24 ± 1.88%) was slight, when compared to the untreated cells (Figure 11). CONT-siRNA did not cause any statistical differences compared to untreated cells. In VEGF-siRNA#1 treated cells, the expression of ANG2 mRNA (41.66 ± 3.03 %, *p* < 0.05, Figure 11A) and protein (59.62 ± 1.84 %, *p* < 0.05, Figure 11B) was significantly reduced compared to untreated cells. siRNA cocktail treated cells (ANG2 mRNA: 39.82 ± 2.78%; protein: 53.86 ± 1.84%) exhibited similar effect with VEGF-siRNA#1 treated cells. In contrast, the result was not reproduced by KSP-siRNA#2, which showed no significant difference in ANG2 expression in HUVECs between KSP-siRNA#2 treated cells and untreated ones (Figure 11).

## Discussion

As tumor cells are characterised by multiple genetic and epigenetic alterations, the single inhibition of one tumour-associated gene as a therapeutic strategy may not be sufficient for inhibition of tumor development. It has been well known that gene therapy targeting either VEGF or KSP alone may cause inhibition of HCC growth [14, 19]. However, the current finding showed that siRNA cocktail silencing VEGF and KSP together could inhibit the proliferation, migration or invasion of HCC cells better than single siRNA simultaneously. On the other hand, the siRNA cocktail might also increase apoptosis induction in HCC cells. This is a better therapeutic strategy which could be adopted in clinics.

As one of the most important angiogenesis-stimulating factors, VEGF is correlated with liver cancer progression through its action of tumor neovascularization, tumor invasion and metastasis [14–16]. Some reports have shown that siRNA-mediated downregulation of VEGF expression results in decreased proliferation and induced apoptosis in colorectal cancer cells [20], prostate cancer cells [21], gastric cancer cells [22]. Our results also demonstrated that siRNA targeting VEGF could inhibit proliferation, migration, invasion and induce apoptosis in hepatocellular carcinoma Hep3B cells. Our observations were consistent with a previous report that also used VEGF-siRNA to suppress VEGF expression in liver cancer cells [14]. To elucidate its molecular mechanisms of VEGF inhibiting cell proliferation and inducing apoptosis, we have examined the expressions of the key regulators Cyclin D1, Bcl-2 and Survivin. Our results demonstrated that the expression levels of Cyclin D1, Bcl-2 and Survivin were significantly decreased in Hep3B cells upon cell transfection with VEGF-siRNA. Cyclin D1 is known to accumulate during the G1 phase of the cell cycle. Overexpression of Cyclin D1 may be a frequent event in hepatocarcinogenesis and therefore plays an important role in growth of liver tumors [23]. In contrast, Bcl-2 and Survivin are thought to be very important anti-apoptotic proteins in cells. They are identified to be one of the mechanisms involved by cancer cells to evade apoptosis. Bcl-2, a prominent member of the Bcl-2 family proteins, is responsible for governing the release of cytochrome c from the mitochondrial membrane, the activation of caspase cascade, the execution of apoptosis, and finally, to the prevention of death in cancer cells [24]. Overexpression of Bcl-2 also may protect human hepatoma cells from antibody mediated apoptosis [25]. Similarly, Survivin, belong to the inhibitors of apoptotic proteins (IAPs), has been implicated in both cell division and inhibition of apoptosis. By inhibiting apoptosis and promoting mitosis, Survivin may confer cancer cell survival and growth. Unlike other members of IAP family, Survivin is lowly or not expressed in normal tissues, but highly in tumor tissues. The induction of apoptosis is generally associated with suppression of Survivin within tumor cells [26]. The overexpression of Survivin in the majority of human tumor types, including liver cancer, can prevent apoptosis by binding and inhibiting pro-apoptotic caspases as a microtubule stabilizer during mitosis, and promote cell cycle progression [27].

In contrast to microtubules which are also presented in post-mitotic cells, KSP is exclusively expressed in mitotic cells, which makes it an important target for anti-mitotics [6]. Therefore, inducing a degradation of KSP by siRNA was expected to lead to a novel approach for the control of cancer cells. In this study, the expression of KSP was downregulated at both mRNA and protein levels in Hep3B cells by KSP-siRNA transfection. This result was similar with reports using KSP-siRNA to monitor the expression of KSP in ovary cancer cells [5], cervical cancer cells, myeloma cells [7], lung carcinoma cells and breast carcinoma cells [8]. In addition, our study also indicated that KSP-siRNA could inhibit proliferation, migration/invasion and induce apoptosis of Hep3B cells. The expression of genes involved in anti-apoptosis (Bcl-2 and Survivin) and proliferation (Cyclin D1) was downregulated in KSP-siRNA transfected cells. From these results, we surmised that the downregulation of Cyclin D1, Bcl-2 and Survivin expressions by VEGF-siRNA or KSP-siRNA transfection in one of the important ways to induce cell apoptosis, subsequently leading cell death.

It has been reported that siRNA cocktail was composed of two different siRNA sequences showed more effective inhibition of the two corresponding target genes at one time than siRNA alone [28]. In present study, we prepared the siRNA cocktail of best siRNAs, analyzed the cell treated with siRNA cocktail and controls, including single siRNA targeting VEGF or KSP and negative control siRNA. Our results revealed that using the siRNA cocktail targeting VEGF and KSP to inhibit the proliferation, migration, invasion and induce apoptosis of Hep3B cells was better than each siRNA alone. This could be explained by the significant downregulation of Cyclin D1, Bcl-2 and Survivin following the treatment of siRNA cocktail as compared to single siRNA simultaneously. Our results corresponded with several previous studies reporting the influences of siRNA cocktail on cell growth and apoptosis of gastric cancer cells [28], pancreatic cancer cells [29] and colorectal cancer cells [30]. The siRNA cocktail exhibited specific and high efficiency on silencing multi genes simultaneously and would have great potential for therapeutic siRNA applications.

Interestingly, we found that VEGF-siRNA exhibited significant inhibition on KSP mRNA and protein levels, while the KSP-siRNA alone did not show any effect on VEGF expression. We assumed that VEGF might be acted as a KSP upstream regulator, which could probably lead to downregulation of KSP expression. The fact that VEGF-siRNA presented similar KSP-siRNA effects on proliferation, migration, invasion and apoptosis of Hep3B cells suggesting that one of inhibiting tumor growth mechanisms of VEGF-siRNA functioned through inhibition of KSP expression. Furthermore, we also found that the expression of KSP protein was suppressed to a greater extent in the siRNA cocktail treated group than that in the KSP-siRNA or VEGF-siRNA alone group, indicating a significant silencing effect of siRNA cocktail on KSP protein expression. The results were supported by the highest inhibition of proliferation, migration, invasion and induction of apoptosis on Hep3B cells by the siRNA cocktail as compared to each of siRNAs alone. In contrast, suppression on VEGF expression in Hep3B cells by the siRNA cocktail was similar to that by the VEGF-siRNA, which supported the observation that KSP-siRNA did not influence VEGF protein expression. This observation was also demonstrated by angiogenesis and ANG2 expression on the HUVECs induced by Hep3B cell culture media following siRNAs treatment. A previous study indicated that HUVECs was induced to form new blood vessels with higher VEGF concentration [31]. In addition, ANG2 which is expressed in the areas undergoing vascular remodeling and leads to decreased vessel maturation and enhanced vessel sprouting could be upregulated by VEGF in endothelial cells [32]. ANG2 inhibition prevents the growth of new vessels by endothelial sprout formation [33]. In our study, the results showed that inhibition of capillary tube-like structure formation and suppression of ANG2 expression in HUVECs by siRNA cocktail was relatively equal when compared to VEGF-siRNA. Meanwhile, KSP-siRNA showed no effect on inhibiting tube formation as well as ANG2 expression in HUVECs. These results demonstrated that VEGF acts as a KSP upstream regulator promoting the effect of KSP on Hep3B cells. More importantly, our estimates were strongly supported by another study reporting a strong upregulation of KSP or Eg5 expression after application of recombinant human VEGF on the differentiated day-13 chick chorio-allantoic membrane (CAM). The results showed that numerous known genes encoding mitotic kinesins were consistently upregulated by VEGF, including KIF4A, KIF11/Eg5, KIF15, KIF20A/Mklp2 and KIF23 [34].

## Conclusion

This study showed that the expression of KSP was inhibited by VEGF-siRNA, suggesting that VEGF serves as an upstream regulator of KSP gene expression. Although there were several reports using siRNA to inhibit VEGF or KSP, combined siRNA therapy to simultaneously reduce VEGF and KSP expression was proved to be an effective approach to inhibit cell growth and induce apoptosis of HCC cells. This could be a new targeted strategy to eradicate HCC cells*.*

## Methods

This experimental study was approved by the Committee for Ethics in Research, University of Science, Vietnam National University.

### Culture of cells

Hep3B cell line (hepatocellular carcinoma cells, HB-8064), HUVECs cell line (human umbilical vein endothelial cells, CRL-1730) were purchased from the American Type Culture Collection (ATCC, Rockville, MD, USA). Hep3B cells were thawed and cultured in DMEM-F12, supplemented with 10% FBS and 0.5% antibiotic-mycotic (All were bought from Sigma-Aldrich, St. Louis, MO, USA). HUVECs cells were thawed and cultured in endothelial cell growth medium-2 (EGM-2) (Lonza, Walkersville, MD, USA), supplemented with 10% FBS and 0.5% antibiotic-mycotic. All cells were maintained at 37°C, 5% CO_2_.

### Transfection of siRNAs

The sequences of the siRNAs targeting VEGF-A (VEGF-A-siRNA, also simply referred to as VEGF-siRNA), siRNAs targeting KSP (KSP-siRNA) and mismatched siRNA (CONT-siRNA) were shown in Table 1. All siRNAs were synthesized by Bioneer (Daejeon, Republic of Korea). Each siRNA was resuspended in nuclease – free water and the stock solutions were stored at 4°C until use. The cells were divided into five groups with different treatments at final concentration of 20 nM: Group 1: untreated cell group, group 2: CONT-siRNA treated cell group, group 3: VEGF-siRNA treated cell group, group 4: KSP-siRNA treated cell group, group 5: siRNA cocktail treated cell group (VEGF-siRNA mixed with KSP-siRNA at equal concentration). All the above siRNAs were transiently transfected with a Lipofectamine RNAiMAX Transfection Reagent kit (Invitrogen Inc., Carlsbad, CA, USA) by reverse transfection protocol. Briefly, for each well of 24-well plate (Corning Costar Corp., Cambridge, MA), 3μl of siRNA duplex (20μM) was mixed with 1 μl transfection reagent and 100 μl Opti-MEM medium supplied with the kit. Then, the siRNA-transfection reagent complex was incubated with 500 μl of diluted cells (5 × 10^4^ cells/ well) for 24-72 hours at 37°C, 5% CO_2_. Control cells (untreated with siRNAs) were also grown under the same condition. The siRNAs treated cells and control cells were harvested during time intervals for transfection efficiency analysis.

### Real-time quantitative reverse transcription PCR (real-time qRT-PCR)

Total RNA was extracted using RNeasy Mini Kit (Qiagen, Valencia, CA, USA). The concentration of RNA was measured using a Biophotometer (Eppendorf, Hamburg, Germany). Real-time qRT-PCR was carried out with a SYBR Green One-Step qRT-PCR kit (Invitrogen Inc., Carlsbad, CA, USA) according to the manufacturer's instructions. The sequences of primers were shown in Table 2. Internal calibration curves were generated by the real time software. A melting curve analysis was carried out between 60°C and 95°C with a plate read every 0.5°C after holding the temperature for 20 seconds. The cycle number (*Ct*) at which the signals crossed a threshold set within the logarithmic phase and the peaks of melting curves were recorded. The relative quantitation of gene expression in terms of fold change was calculated using the 2^-ΔΔCt^ method [35]. Relative expression levels of the target genes in each treatment group were derived from normalizing the Ct value of the target genes against that of an endogenous reference (β-actin) and a calibrator (control cells).

**Table 2:** **Sequences of the primers for Real-time qRT-PCR**

Gene	Sequences (5’–3’)	Product size (bp)
VEGF	F: CCATGAACTTTCTGCTGTCTT	250
	R: ATCGCATCAGGGGCACACAG	
KSP	F: CTGAACAGTGGGTATCTTCCTTA	480
	R: GATGGCTCTTGACTTAGAGGTTC	
Cyclin D1	F: GCCCGAGGAGCTGCTGCAAA	358
	R: CCTGGCGCAGGCTTGACTCC	
Bcl-2	F: CGGTGCCACCTGTGGTCCAC	174
	R: TCCCCCAGTTCACCCCGTCC	
Survivin	F: GGACCGCCTAAGAGGGCGTGC	145
	R: AATGTAGAGATGCGGTGGTCCTT	
ANG2	F: TGGGATTTGGTAACCCTTCA	234
	R: GTAAGCCTCATTCCCTTCCC	
β-actin	F *:* ACACTGTGCCCATCTAGGAGG	680
	R: AGGGGCCGGACTCGTCATACT	

F and R are Forward and Reverse, respectively.

### Western blot

After washing with cold PBS, the cells were lysed by a lysis buffer containing 0.01M Tris, pH 7.5, 0.1M NaCl, 1% Triton X-100, 0.5% sodium deoxycholate, and 0.1% sodium dodecyl sulfate (SDS), with added protease inhibitors. Total proteins in cell lysates were separated by 10% SDS-polyacrylamide gel electrophoresis (PAGE) and transferred to a polyvinylidene fluoride (PVDF) blotting membrane (Sigma-Aldrich, St. Louis, MO, USA). The membranes were blocked in blocking solution BSA (Sigma-Aldrich, St. Louis, MO, USA) and incubated with mouse anti-Eg5/KSP monoclonal antibody (1:200), mouse anti-VEGF monoclonal antibody (1:200), mouse anti-Cyclin D1 monoclonal antibody (1:500), mouse anti-Bcl-2 monoclonal antibody (1:500), mouse anti-Survivin monoclonal antibody (1:500), mouse anti-ANG2 monoclonal antibody (1:200) (All were bought from Abcam, Cambridge, ENG, UK) for 1 hour at room temperature. After washing, the membranes were incubated for 45 minutes with horseradish peroxidase (HRP)-linked goat anti-mouse IgG (1:5000, Sigma-Aldrich, St. Louis, MO, USA). The protein bands were visualized by enhanced chemiluminescence (Sigma-Aldrich, St. Louis, MO, USA). Mouse monoclonal Ab against β-actin (Abcam, Cambridge, ENG, UK) was used as a housekeeping gene control. Band intensities were semi-quantitatively analyzed by Image J densitometer (NIH, Bethesda, MD, USA).

### Enzyme linked immunosorbent assay (ELISA)

The amount of VEGF in cell supernatants was measured by using human VEGF ELISA kit (Life Technologies, Carlsbad, CA, USA) following the manual of the kit. The human VEGF ELISA kit is a “sandwich” enzyme immunoassay employing monoclonal and polyclonal antibodies. Quantitation can be determined by constructing an absolute standard curve using known concentrations of human VEGF proteins.

### Cell proliferation assay

Cell proliferation was measured by WST-1 assay kit (Roche, Basel, Switzerland). Briefly, siRNAs transfected cells and control cells were seeded at a concentration of 3 × 10^3^ cells per well in 96-well plates (Corning Inc., NY, USA). For indicated time, WST-1 solution was applied at 10 μl per well and incubated for 4 hours at 37°C, 5% CO_2_. The absorbance was measured with a microplate ELISA reader (BioTek, Winooski, VT, USA) at 450 nm.

### Clonogenic survival assay

Cells were seeded at a density of 500 cells per well in 6-well plates (Corning Inc., NY, USA) contained complete medium followed by treatment with siRNAs. The medium was then replaced with fresh medium and incubated for an additional 10 days. Clones were fixed with 4% paraformaldehyde for 30 minutes and stained with Crystal Violet (Sigma-Aldrich, St. Louis, MO, USA) for about 15 minutes. Stained clones that had more than 50 cells were counted at low magnification.

### Wound-healing assay

Cell migration was measured by wound-healing assay. Hep3B cells were seeded and transfected with siRNAs as described above in 24-well plates (Corning Inc., NY, USA) at the density of 5 × 10^4^ cells per well. After 48 hours, wound was made through confluent monolayer cells with a pipette tip. Wounded monolayers were then washed with PBS, and incubated in DMEM-F12 without FBS. Photographs of cells were taken at 0, 24, 48, and 72 hours to monitor cell movements.

### Transwell invasion assay

Cell invasion was carried out by transwell assays. The upper surface of the transwell filters (Corning Inc., NY, USA) was coated with matrigel (BD Biosciences, Franklin Lakes, NJ, USA). The siRNAs treated cells and control cells (1 × 10^5^ cells) were suspended in 200 μl serum-free media, and then added to the chamber. The chamber afterthat was placed in a 24-well plate containing complete medium. After 48 hours of incubation at 37°C, the filters were gently taken out and matrigel on the upper surface of the filters was removed by cotton swabs. Cells on the underside of transwell filters were fixed with 4% paraformaldehyde for 30 minutes, stained with Crystal Violet (Sigma-Aldrich, St. Louis, MO, USA) for 10 minutes, and then photographed. For quantitative assessment, the number of invading cells was counted from five randomly selected visual fields per filter.

### Apoptosis assay

Apoptosis was investigated by flow cytometry using annexin V and propidium iodide (PI) (BD Biosciences, Franklin Lakes, NJ, USA). Briefly, the cell concentration was firstly adjusted to 1 × 10^6^ cells/ml, and then 1 ml of the cell suspension was taken and centrifuged at 500 × g for 10 minutes at 4°C. The pellet was rinsed twice with PBS and then re-suspended in a proper volume of binding buffer so that the cell concentration was 5 × 10^4^ cells/ml. After addition of 10 μl Annexin V-FITC and 5 μl PI followed by gentle mix, a 15 minute reaction was initiated at room temperature in darkness. After that, 300 μl binding buffer was added and flow cytometry (FACSCalibur) using CellQuest Pro software (BD Biosciences, Franklin Lakes, NJ, USA) was performed to detect cell apoptosis rate (%).

### Tube formation assay

The ability of endothelial cells to sprout new blood vessels stimulated by pro-angiogenic factors released from Hep3B cells was examined in HUVECs angiogenesis *in vitro* model. Briefly, 6 × 10^4^ HUVECs were collected, resuspended in a conditioned medium, which was the supernatant of siRNAs treated Hep3B cells, seeded in 24-well plates coated with 100 μl Matrigel (BD Biosciences, Franklin Lakes, NJ, USA), and cultured at 37°C, 5% CO_2_. After incubation for 24 hours, numbers of branching points were counted.

### Statistical analysis

Each experiment was performed in triplicate for all data (n = 3). Data was expressed as mean ± standard error of the mean. Statistical comparisons were performed using the Student’s *t* –test and ANOVA. *p* -values < 0.05 were considered to be statistically significant.

## Abbreviations

BSA: Bovine serum albumin
DAPI: 4',6-diamidino-2-phenylindole
EtdBr: Ethidium bromide
FITC: Fluorescein isothiocyanate
HCC: Hepatocellular carcinoma
HRP: Horseradish peroxidase
IAP: Inhibitors of the apoptosis protein
KSP: Kinesin spindle protein
mRNA: Messenger RNA
PAGE: Polyacrylamide gel electrophoresis
PI: Propidium iodide
PVDF: Polyvinylidene fluoride
Real-time qRT-PCR: Real-time quantitative reverse transcription PCR
RNAi: RNA interference
RT-PCR: Reverse transcription PCR
SDS: Sodium dodecyl sulfate
siRNA: Small interfering RNA
VEGF: Vascular endothelial growth factor.
